# A Bayesian Inference Based Computational Tool for Parametric and Nonparametric Medical Diagnosis

**DOI:** 10.3390/diagnostics13193135

**Published:** 2023-10-05

**Authors:** Theodora Chatzimichail, Aristides T. Hatjimihail

**Affiliations:** Hellenic Complex Systems Laboratory, Kostis Palamas 21, 66131 Drama, Greece; tc@hcsl.com

**Keywords:** Bayesian diagnosis, Bayesian inference, prior probability, posterior probability, likelihood, parametric distribution, nonparametric distribution, copula distribution, kernel density estimator, probability density function, diabetes mellitus

## Abstract

Medical diagnosis is the basis for treatment and management decisions in healthcare. Conventional methods for medical diagnosis commonly use established clinical criteria and fixed numerical thresholds. The limitations of such an approach may result in a failure to capture the intricate relations between diagnostic tests and the varying prevalence of diseases. To explore this further, we have developed a freely available specialized computational tool that employs Bayesian inference to calculate the posterior probability of disease diagnosis. This novel software comprises of three distinct modules, each designed to allow users to define and compare parametric and nonparametric distributions effectively. The tool is equipped to analyze datasets generated from two separate diagnostic tests, each performed on both diseased and nondiseased populations. We demonstrate the utility of this software by analyzing fasting plasma glucose, and glycated hemoglobin A1c data from the National Health and Nutrition Examination Survey. Our results are validated using the oral glucose tolerance test as a reference standard, and we explore both parametric and nonparametric distribution models for the Bayesian diagnosis of diabetes mellitus.

## 1. Introduction

Medical diagnosis is a critical process of accurately identifying pathological conditions in patients. The term “diagnosis” has its etymological origins in the ancient Greek word “διάγνωσις”, signifying “discernment” [1]. Traditionally, diagnostic tests are used to divide individuals into two principal categories: those who are afflicted with a specific disease and those who are not. Notably, the probability distributions associated with quantitative diagnostic test outcomes often demonstrate some overlap between the diseased and nondiseased groups. To address this, numerical diagnostic thresholds or cut-off points have been formulated to provide a binary classification of these test outcomes [2]. Nevertheless, this introduces a certain measure of uncertainty into the diagnostic accuracy of those tests [3]. This dichotomous method represents a significant shift in medical decision-making by linking a continuum of evidence to binary clinical decisions such as to treat or not to treat [4].

Despite the evident efficiency of traditional diagnostic methods, they sometimes fail to capture the complexity and heterogeneity of disease presentations across diverse populations [5]. To address these limitations, our research focuses on implementing Bayesian inference to calculate the posterior probabilities associated with disease diagnosis [6,7,8,9]. Within this Bayesian paradigm, prior probabilities of disease are integrated with distributions of diagnostic measurands in both diseased and nondiseased populations. This approach enables the evaluation of the information conveyed via diagnostic measurements and the combination of data from multiple diagnostic tests, which may improve diagnostic accuracy and precision while introducing flexibility, adaptability, and versatility into the diagnostic process [10]. Furthermore, the Bayesian approach extends its utility beyond the medical field by offering a robust framework for quantifying uncertainty in various domains, thereby enriching its applicability in both diagnostic and prognostic contexts [11,12].

A considerable challenge in integrating Bayesian inference into medical diagnosis is the limited availability of literature detailing the statistical distributions of diagnostic variables in both pathological and non-pathological states [13].

The ubiquitous application of the normal distribution in clinical laboratory indicators is due, in part, to its mathematical simplicity, the foundational Central Limit Theorem, and a rich collection of statistical methods designed for Gaussian data [14]. However, the universal applicability of the normal distribution is subject to critique, especially when dealing with clinical measurands that exhibit skewness, bimodality, or multimodality [15]. Hence, while the normal distribution remains invaluable in statistical modeling, critical evaluation of its appropriateness for specific diagnostic measurands is necessary. This evaluation should be accompanied by an openness to adopt alternative statistical distributions when needed [16].

This foundational data is crucial for Bayesian inference, establishing the essential context against which new diagnostic measurements can be compared. The absence of such normative data could potentially compromise the reliability and validity of Bayesian diagnostic methods.

To address the complex issues related to Bayesian diagnosis and the selection of appropriate statistical distributions for diagnostic variables, we have developed the *Bayesian Diagnosis* program, an interactive software tool programmed in the Wolfram Language. This tool allows users to explore and compare both parametric and nonparametric distributions to calculate posterior probabilities for disease. It is designed to analyze datasets of measurements of two distinct diagnostic tests, performed on both diseased and nondiseased populations.

## 2. Methods

### 2.1. The Program

**Figure 1:**
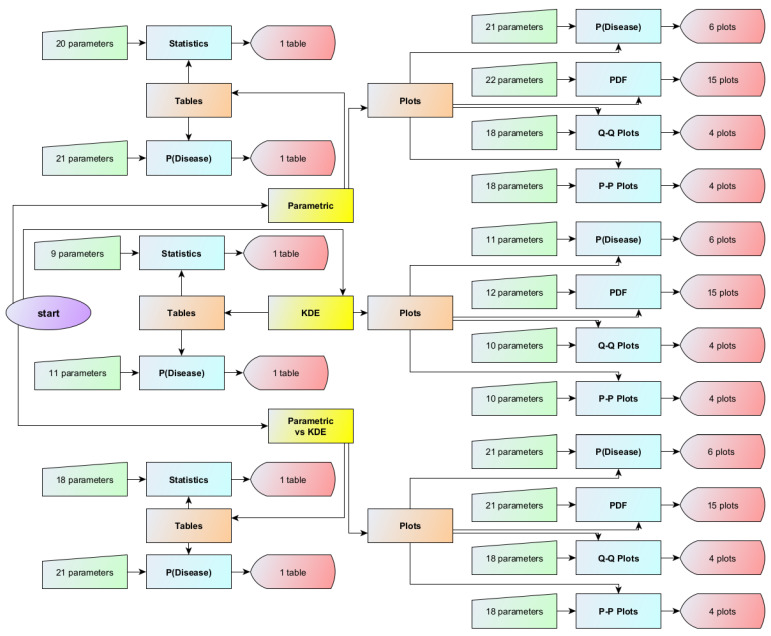
The flowchart of the *Bayesian Diagnosis* program.

*Bayesian Diagnosis* was developed using Wolfram Mathematica^®^ Ver. 13.3 (Wolfram Research, Inc., Champaign, IL, USA (2023)). This interactive program consists of three primary modules with eighteen submodules. It allows the calculation, plotting, and comparison of Bayesian posterior probabilities of disease for two diagnostic tests, assuming two sets of alternative parametric and nonparametric distributions of the measurements of those tests in diseased and nondiseased populations (refer to Figure 1 and to Supplementary File S1). It is freely available as a Wolfram Notebook (.nb) (Supplementary File: BayesianDiagnosis.nb). It can be run on Wolfram Player^®^ or Wolfram Mathematica^®^ (refer to Appendix B).

#### Datasets

As datasets are considered tuples of data. Although the program includes four datasets of measurements, one for each diagnostic test, applied to a diseased and a nondiseased population, these can be replaced by other appropriate datasets selected by the user (refer to Appendix B). Therefore, it can be used for any combination of diagnostic tests and diseases.

### 2.2. Computational Methods

#### 2.2.1. Bayesian Diagnostic Approach

The Bayesian diagnostic approach is a cornerstone in statistical inference and particularly useful in medical diagnosis [6,17,18]. The approach relies on Bayes’ theorem [7]. For effective implementation of the Bayesian diagnostic method, knowledge concerning the statistical distributions of the measurements of the diagnostic tests is essential [14].

Bayes theorem is presented in Appendix A.

#### 2.2.2. Parametric Distributions

Parametric statistics assume that dataset data comes from a population that can be adequately modeled with a probability distribution that has a fixed set of parameters [19]. The parametric distributions provided by the program are the following:

1. Normal Distribution
  1.1 Univariate
  1.2 Bivariate
2. Lognormal Distribution
  2.1 Univariate
  2.2 Bivariate
3. Gamma Distribution
  3.1 Univariate
  3.2 Bivariate
4. Copula Distributions

The copula distributions of the program are bivariate, with a bivariate normal distribution with correlation *ρ* as kernel, and univariate normal, lognormal and gamma marginals.

The probability density functions (PDFs) of the parametric distributions are mathematically defined in Appendix A.

#### 2.2.3. Nonparametric Distributions

Conversely, nonparametric models were also employed, which do not make a priori assumptions about the distribution’s mathematical form [20]. These are particularly useful for exploratory data analysis and are implemented as shown in Appendix A.

##### Histograms

A histogram is the graphical representation of the distribution of a dataset as a series of bins.

The program plots histograms of the provided datasets.

##### Kernel Density Estimators (KDEs)

In contrast to histograms, a KDE generates a continuous and smooth estimate of the underlying PDF by summing the contributions of kernel functions centered at each data point.

KDEs offer a flexible nonparametric approach to density estimation, allowing for a better representation of the underlying distribution of the data.

The program provides univariate and bivariate Gaussian KDEs. The bivariate KDEs use radial-type kernels.

### 2.3. Interface of the Program

**Figure 2:**
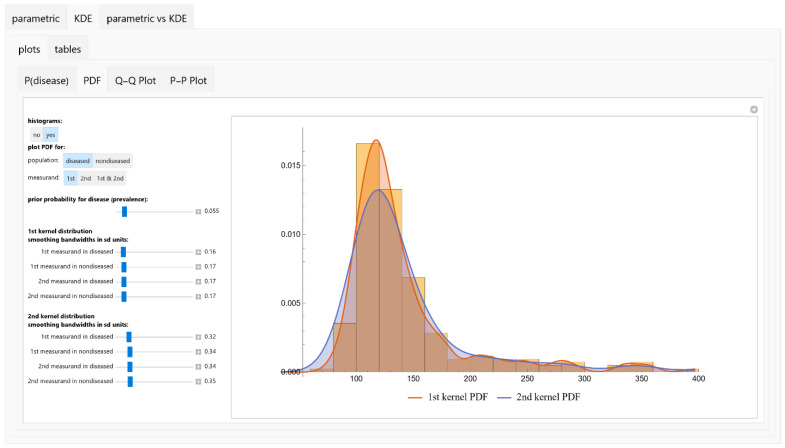
Screen shot of the *Bayesian Diagnosis* program.

The *Bayesian Diagnosis* program is equipped with an intuitive tabbed user interface (refer to Figure 2). This design facilitates seamless navigation through its various modules and submodules. Users have the capability to input and modify a range of parameters, including prior probabilities and measurement parameters. Additionally, the interface allows for the selection of both parametric distributions and KDEs pertinent to medical diagnosis (refer to Appendix C and Supplementary File S1).

#### 2.3.1. Input Parameters

##### Prior Probability

The user initiates the diagnostic evaluation by specifying the prior probability of disease occurrence in the population under study. This serves as a foundational measure for subsequent analyses.

##### Parametric Distributions

To facilitate a diagnostic model, the program allows for the definition of various parametric distributions for both the diseased and nondiseased populations across two diagnostic tests.

1. Distribution Selection: The user selects the type of distribution from a predefined list:
  1.1 Normal Distribution.
  1.2 Lognormal Distribution.
  1.3 Gamma Distribution.
2. Statistical Parameters: For each chosen distribution, the user defines the mean *μ* and standard deviation *σ* of the measurand in the respective population.
3. Correlation Coefficients: The user specifies the correlation coefficients *ρ* between the measurands of the first and second diagnostic tests for both diseased and nondiseased populations.

##### KDEs

Alternatively, the user can opt to define the KDEs for the measurands in both diseased and nondiseased populations across the two tests:

1. Bandwidth Parameter: For each KDE, the user defines the bandwidth parameter *h*.
2. Correlation Coefficients: As with parametric distributions, the user defines the correlation coefficients *ρ* between the measurands of the two diagnostic tests.

#### 2.3.2. Output Specifications

##### Visualizations

The program generates a series of plots designed to elucidate various diagnostic metrics and statistics:

1. Posterior Probability of Disease: Plots are generated to show the posterior probability of disease for each measurand and their combination.
2. PDFs: Univariate PDFs for each measurand and the bivariate PDF of their combination are plotted. An option to overlay histograms on these plots is also provided.
3. Quantile–Quantile (Q–Q) Plots: These plots are produced for each measurand to examine its distributional characteristics [21].
4. Probability–Probability (P–P) Plots: Similar to Q–Q plots, P–P plots are generated for further assessment of the distribution of each measurand [21].

The descriptions of the Q–Q and P–P plots are presented in the Supplementary File S2.

##### Tables

1. Population Statistics: The program tabulates key statistical metrics such as mean, median, standard deviation, skewness, kurtosis, and prior probability for each user-defined distribution and dataset. For each bivariate distribution of the two measurands in diseased and nondiseased populations, the correlation coefficients are calculated and displayed.
2. Posterior Disease Probabilities: For a user-defined pair of test measurement values, the program computes and presents the posterior probabilities for disease for each measurand and their combination.

By providing this comprehensive set of input parameters and output specifications, the program offers a robust platform for exploring the Bayesian diagnosis of disease using either parametric distributions or KDEs of medical diagnostic measurands.

#### 2.3.3. Illustrative Application

To demonstrate the application of the program, fasting plasma glucose (FPG) was used as the first measurand and glycated hemoglobin A1c (HbA1c) as the second measurand for Bayesian diagnosis of diabetes mellitus. The oral glucose tolerance test (OGTT) was used as the reference diagnostic method. A diagnosis of diabetes was confirmed if the plasma glucose (PG) value was equal to or exceeded 200 mg/dL, measured two hours after oral administration of 75 g of glucose [22], during an OGTT (2-h PG). It is noteworthy that the study population was confined to individuals aged between 40 and 60 years, a decision informed by the well-documented strong correlation between age and the prevalence of diabetes [23].

National Health and Nutrition Examination Survey (NHANES) data from participants was retrieved for the period from 2005 to 2016 [24] (*n* = 60,936). NHANES is a series of studies designed to evaluate the health and nutritional status of adults and children in the United States.

The inclusion criteria for participants were:

1. Age 40–60 years (*n* = 11,782);
2. Valid FPG, HbA1c, and OGTT measurements (*n* = 4015);
3. A negative response to NHANES question DIQ010 regarding a diabetes diagnosis [25] (*n* = 3854);
4. Non-pregnancy status (*n* = 3854).

Participants with a 2-h PG measurement of ≥ 200 mg/dL were considered diabetic (*n* = 211).

Descriptive statistics, including the mean, median, and standard deviation, were computed for each dataset and correlation coefficients for their combination. Univariate distributions were employed to model the distributions of FPG and HbA1c and bivariate distributions to model the joint distribution of FPG and HbA1c. Likelihoods and posterior probabilities were estimated for FPG, HbA1c and their combination.

The prior probability of diabetes was estimated as follows:

$$v=\frac{211}{3854}=0.055$$

The statistics of the dataset are presented in Table 1.

## 3. Results

Using the settings of Table 2, the program generated the plots of Figure 3, Figure 4, Figure 5, Figure 6, Figure 7, Figure 8, Figure 9, Figure 10, Figure 11, Figure 12 and Figure 13 and the tables of Figure 14 and Figure 15.

The KDEs smoothing bandwidth was set to double that given with Silverman’s rule of thumb [26,27].

**Figure 3:**
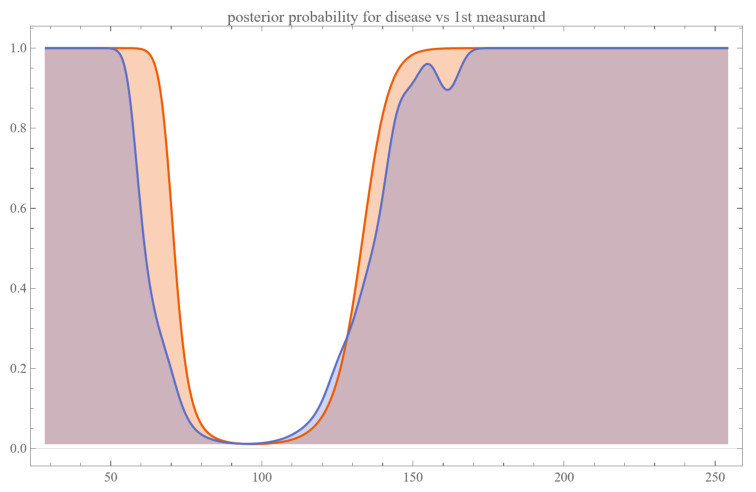
Posterior probability of disease (diabetes) versus the first measurand (FPG), assuming parametric and KDE distributions of the measurand, with the settings of the program in Table 2.

**Figure 4:**
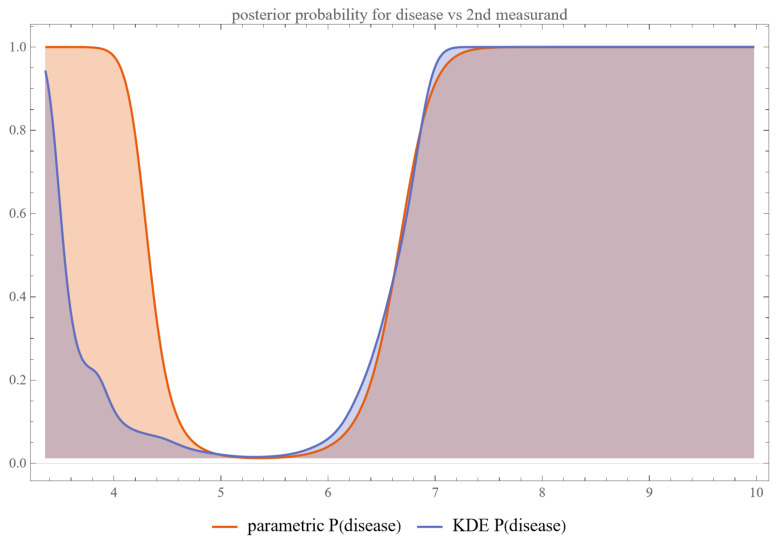
Posterior probability of disease (diabetes) versus the second measurand (HbA1c), assuming parametric and KDE distributions of the measurand, with the settings of the program in Table 2.

Figure 3 and Figure 4 show the plots of the posterior probability of diabetes versus FPG and HbA1c, respectively. The curves of the parametric distributions are smooth double sigmoidal, while the curves of the nonparametric distributions are multimodal.

**Figure 5:**
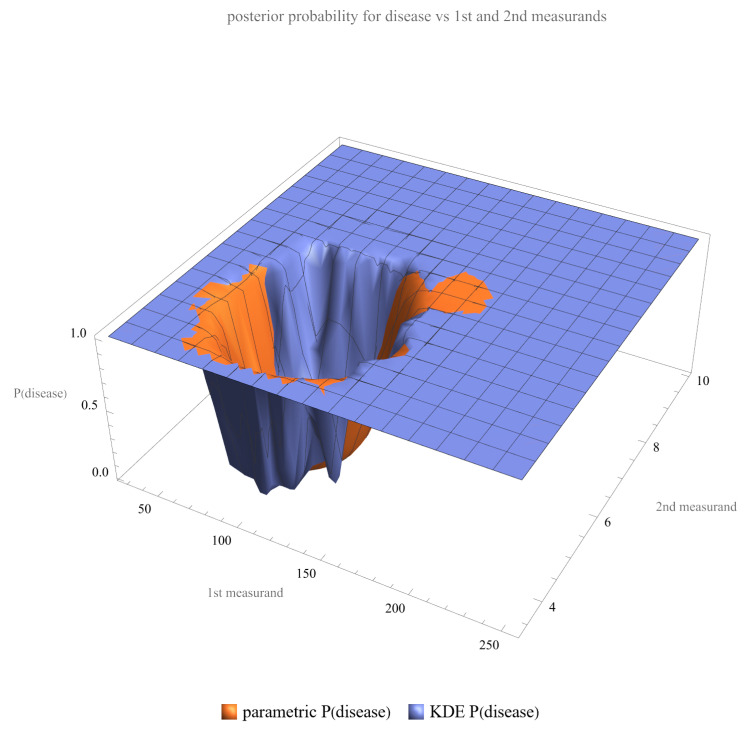
Posterior probability of disease (diabetes) versus both measurands (FPG and HbA1c), assuming parametric and KDE distributions of the measurands, with the settings of the program in Table 2.

Figure 5 shows the plot of the posterior probability of diabetes versus FPG and HbA1c combined. The surface of the parametric distribution is smooth, while the surface of the nonparametric distribution is multimodal.

**Figure 6:**
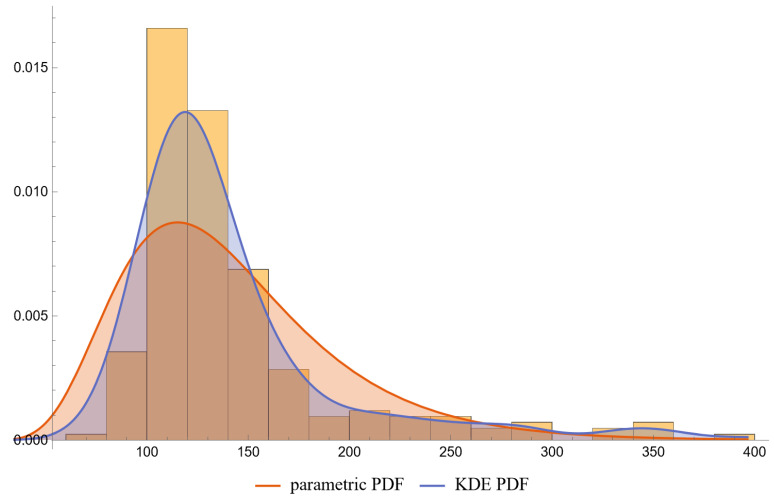
The PDF of the first measurand (FPG) in diseased (diabetic patients), assuming parametric and KDE distributions of the measurand, and the histogram of the respective dataset (NHANES dataset), with the settings of the program in Table 2.

**Figure 7:**
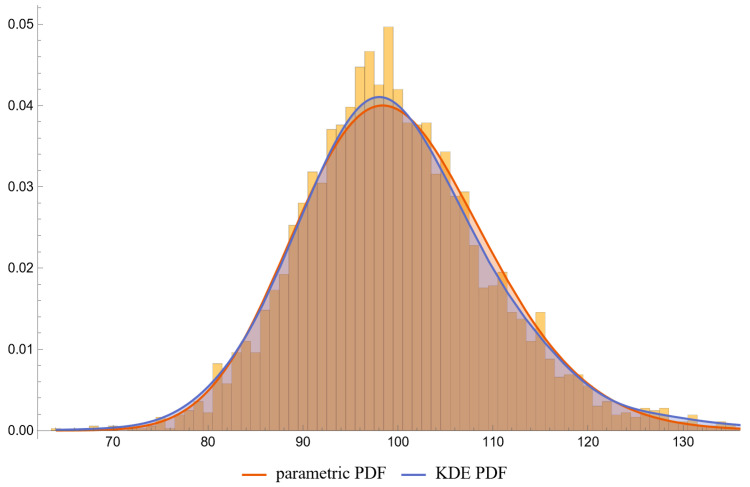
The PDF of the first measurand (FPG) in nondiseased (nondiabetic patients), assuming parametric and KDE distributions of the measurand, and the histogram of the respective dataset (NHANES dataset), with the settings of the program in Table 2.

**Figure 8:**
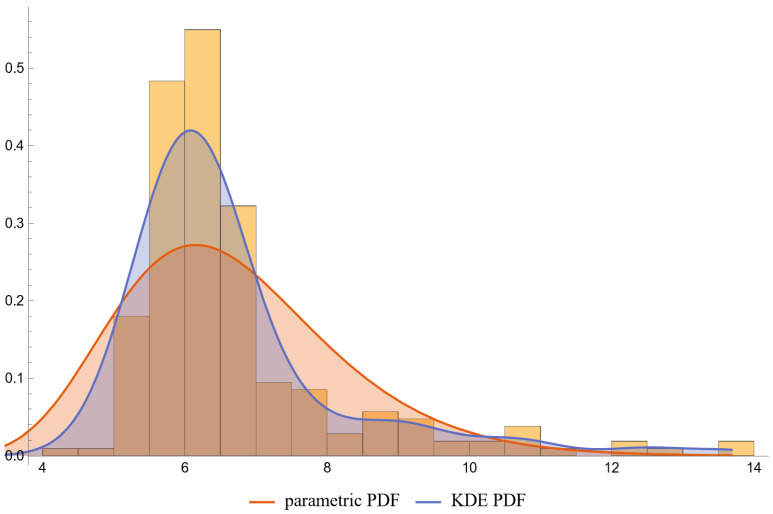
The PDF of the second measurand (HbA1c) in diseased (diabetic patients), assuming parametric and KDE distributions of the measurand, and the histogram of the respective dataset (NHANES dataset), with the settings of the program in Table 2.

**Figure 9:**
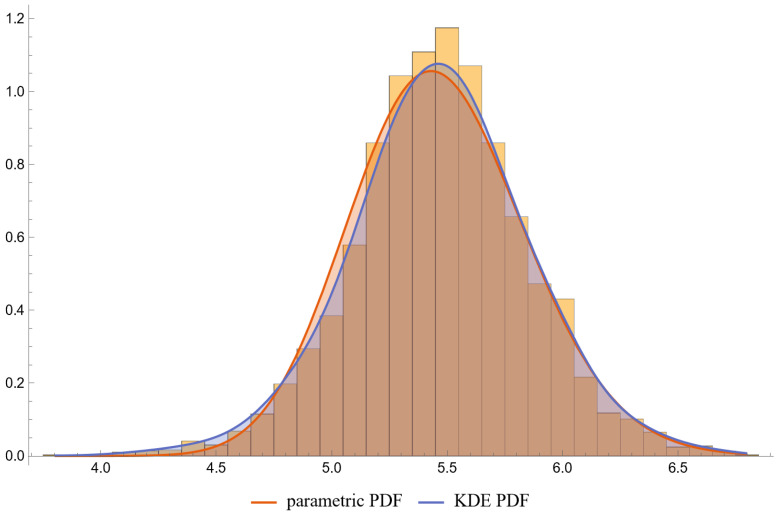
The PDF of the second measurand (HbA1c) in nondiseased (nondiabetic patients), assuming parametric and KDE distributions of the measurand, and the histogram of the respective dataset (NHANES dataset), with the settings of the program in Table 2.

Figure 6, Figure 7, Figure 8 and Figure 9 show the PDF of FPG and HbA1c in diabetic and nondiabetic patients and the histograms of the respective NHANES datasets. It is visually evident that the nonparametric distributions fit the datasets better, especially in diabetic patients.

**Figure 10:**
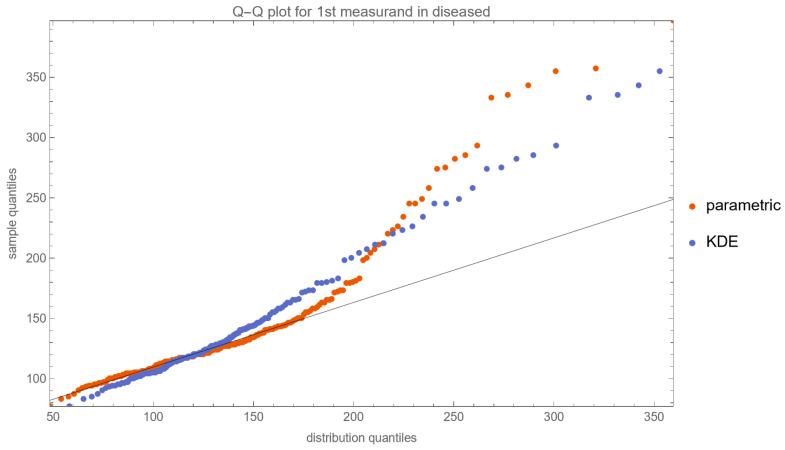
The Q–Q plot of the first measurand (FPG) in diseased (diabetic patients) versus the respective dataset (NHANES dataset), assuming parametric and KDE distributions of the measurand, with the settings of the program in Table 2.

**Figure 11:**
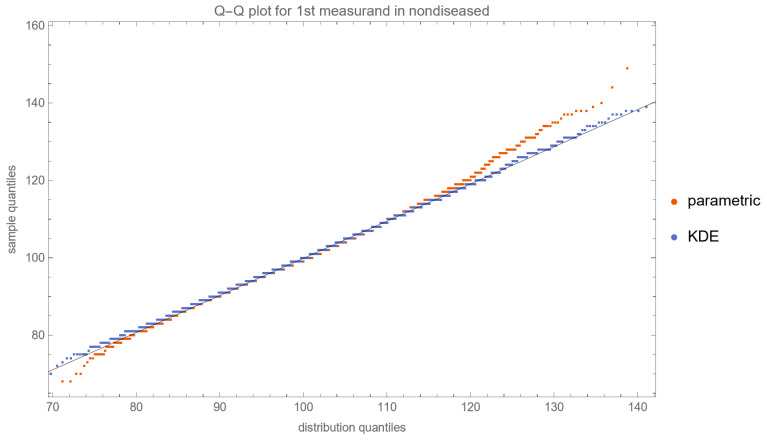
The Q–Q plot of the first measurand (FPG) in nondiseased (nondiabetic patients) versus the respective dataset (NHANES dataset), assuming parametric and KDE distributions of the measurand, with the settings of the program in Table 2.

**Figure 12:**
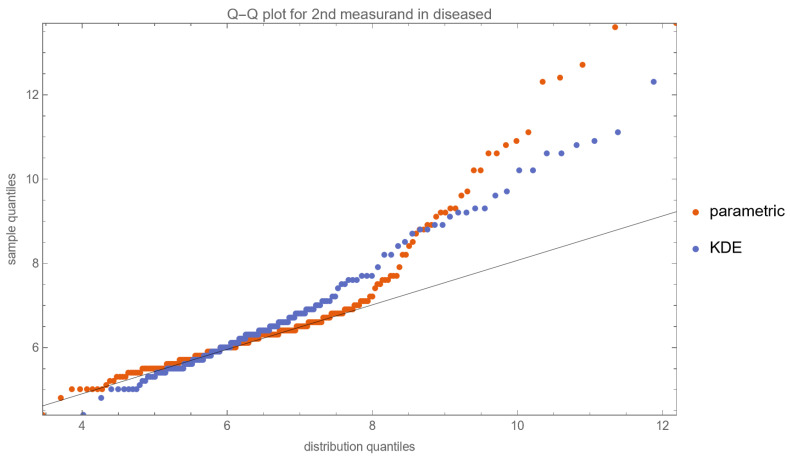
The Q–Q plot of the second measurand (HbA1c) in diseased (diabetic patients) versus the respective dataset (NHANES dataset), assuming parametric and KDE distributions of the measurand, with the settings of the program in Table 2.

**Figure 13:**
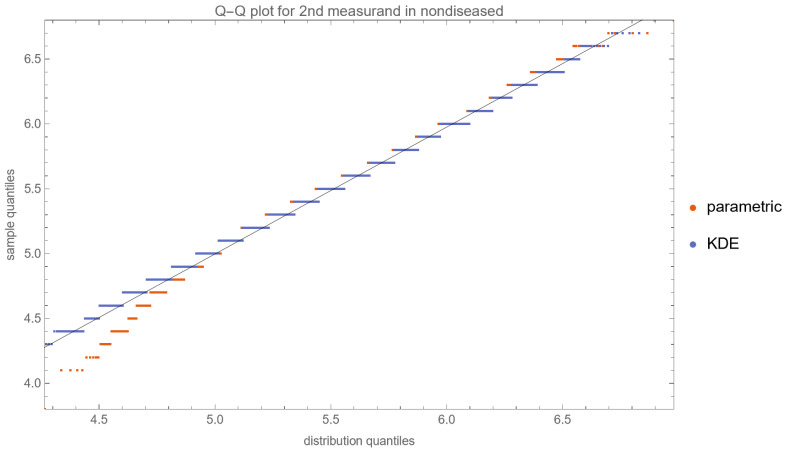
The Q–Q plot of the second measurand (HbA1c) in nondiseased (nondiabetic patients) versus the respective dataset (NHANES dataset), assuming parametric and KDE distributions of the measurand, with the settings of the program in Table 2.

Figure 10, Figure 11, Figure 12 and Figure 13 show the Q–Q plots of the parametric and KDE distributions of FPG and HbA1c in diabetic and nondiabetic patients versus the respective NHANES datasets. The plots show clearly that the nonparametric distributions fit the datasets better, especially in diabetic patients.

**Figure 14:**
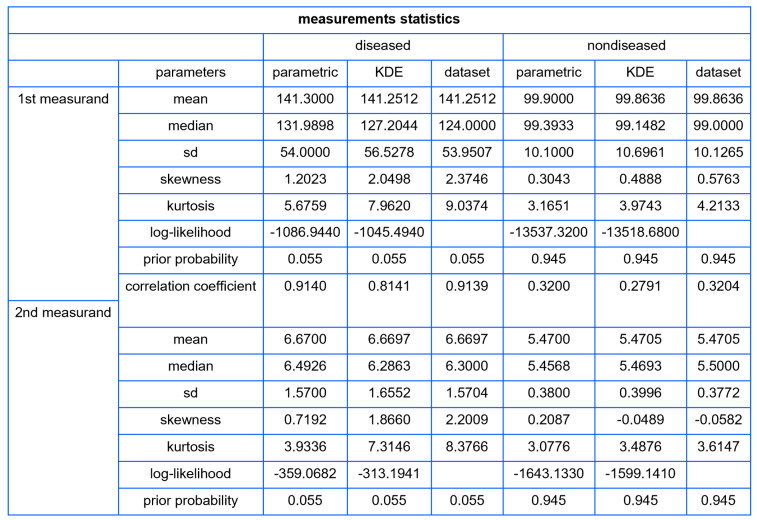
Descriptive statistics of the distributions of the measurands (FPG and HbA1c) in diseased (diabetic patients) and nondiseased (nondiabetic patients), assuming parametric and KDE distributions, and of the respective datasets (NHANES datasets), with the settings of the program in Table 2.

Figure 14 shows a table with the descriptive statistics of FPG and HbA1c in diabetic patients and nondiabetic patients, assuming parametric and KDE distributions, and of the respective NHANES datasets. The data, including the loglikehood values, supports the hypothesis that the nonparametric distributions fit the datasets better, especially in diabetic patients.

**Figure 15:**
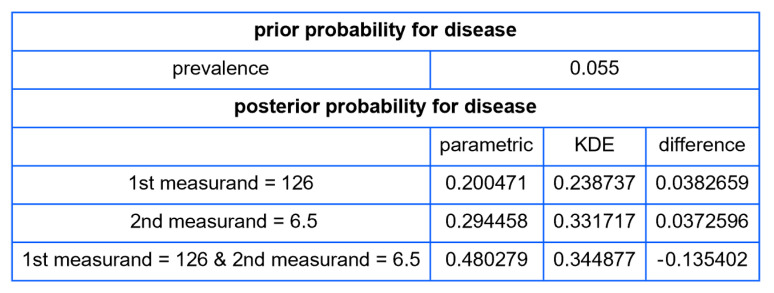
The prior and posterior probabilities of disease (diabetes) for values of the first measurand (FPG) equal to 126 mg/dL and of the second measurand (HbA1c) equal to 6.5%, assuming parametric and KDE distributions, with the settings of the program in Table 2.

Figure 15 shows a table of prior and posterior probabilities for disease (diabetes) for values of FPG equal to 126 mg/dL and of HbA1c equal to 6.5%, the established thresholds of the two measurands for the diagnosis of diabetes [22], assuming parametric and KDE distributions.

## 4. Discussion

### 4.1. Reevaluation of Traditional Diagnostic Methods

The findings of the present study highlight the importance of considering incorporating Bayesian methods in medical diagnosis and management. Conventional approaches based on rigid diagnostic criteria are often unable to account for the intricate relationships between disease pathology and diagnostic procedures thus limiting personalized patient care options. [28]. In stark contrast, Bayesian methodologies offer a framework that enhances diagnostic precision through a more comprehensive probabilistic assessment [5]. This Bayesian foundation, therefore, could serve as an enabler for tailored medical interventions, echoing similar arguments in existing literature advocating for individualized medicine [29].

The study population was confined to individuals aged between 40 and 60 years. This restriction allowed for a more homogeneous prior probability, thereby reducing the impact of age-specific variations in the prevalence of the condition under study.

Even though the KDEs from our illustrative application, as parameterized in Table 2, provide only an approximate fit to the NHANES datasets for FPG and HbA1c measurements, the posterior probabilities for diabetes delineated in Figure 15 suggest a limited concordance between the classification criteria of diabetes derived from the OGTT, HbA1c, and FPG tests [22], as found previously in existing literature [30].

### 4.2. Challenges and Considerations in Bayesian Analysis for Disease Diagnosis

Despite the evident merits of Bayesian analytics in medical diagnostics, it is paramount to address the intrinsic challenges associated with this methodological shift. One such issue resides in the limited availability of scholarly publications that provide a comprehensive statistical exploration of the measurands in both the diseased and nondiseased populations [31].

#### 4.2.1. Ramifications of Incomplete Information

1. Over-dependence on Prior Probabilities: The scarcity of empirically derived distributions amplifies reliance on prior probabilities, thereby inducing distortions in the calculation of posterior probabilities. This could result in suboptimal clinical judgments and potentially inaccurate diagnoses [32].
2. Elevated Uncertainty: Insufficient data contributes to broader confidence intervals in the computed posterior probabilities, which, in turn, could exacerbate clinical indecisiveness [33].
3. Risk of Bias: The introduction of systemic bias due to unrepresentative datasets could compromise the fidelity of Bayesian calculations [7].
4. Imperative for Collaborative Research: More coordinated research is needed, including multi-center studies, meta-analyses, and open-access databases—to accumulate and disseminate data essential for effective Bayesian diagnosis [34].
5. Exploration of Alternative Methodologies: Given the lack of comprehensive data, the utility of combining Bayesian methods with other statistical and computational techniques or diagnostic modalities becomes increasingly pertinent [35,36].

#### 4.2.2. Parametric Versus Nonparametric Bayesian Models

In the context of diagnosing diabetes mellitus through FPG and HbA1c levels, our computational tool revealed that nonparametric Bayesian models typically produce a better fit to data distributions, corroborating existing literature that emphasizes the robustness of nonparametric techniques in capturing complex data distributions [26,37].

#### 4.2.3. Multimodal Versus Double Sigmoidal Bayesian Probability of Disease Curve

The nonparametric Bayesian probabilities for disease exhibited multimodal patterns, in contrast to the bimodal, double sigmoidal curves generated by parametric models.

##### Multimodal Curve

Potential Causes:

(a) Complex Pathophysiology: Multiple etiological pathways may influence the same measurand in divergent ranges, adding layers of complexity to diagnostic processes [13].
(b) Diagnostic Confounders: External variables affecting the measurand could compromise its efficacy as a standalone diagnostic criterion [38].
(c) Population Subgroups: The existence of demographically or genetically distinct subgroups within the studied population could also account for the observed multimodality [39].
(d) Statistical Artifacts: Demographically or genetically distinct subgroups may be a factor contributing to observed multimodal distributions [39].

Theoretical Implications:

Multimodal distributions present a clinical conundrum, compelling healthcare providers to potentially employ additional diagnostic tests or even alternative methodologies [13].

##### Double Sigmoidal Curve

A curve composed of two mirrored sigmoid functions, one delineating the probability behavior for lower measurand values and the other for higher values— presents a compelling intricacy in the realm of diagnostic statistics and medical decision-making.

Interpretation

(a) Two Zones of Risk: Such a curve suggests that the risk of the disease is heightened both at low and high extremes of the measurand but reduced in a middle “safe zone.”
(b) Multifactorial Etiology: This might reflect a situation where both deficiency and excess of a particular biological factor contribute to disease risk. For example, both low and elevated levels of hormones may pose challenges to physiological homeostasis.

Clinical and Diagnostic Implications

(a) Threshold Decision-making: Unlike a single sigmoid curve, where one threshold may be adequate for diagnosis, the double sigmoid may necessitate multiple thresholds, defining a “safe zone” for the measurand.
(b) Treatment Strategies: Clinicians must be cautious when intervening based on such a measurand, as moving the measurand too far in either direction could heighten risk.
(c) Population Stratification: This curve shape might imply that different sub-populations or disease subtypes could be better distinguished with additional tests or measurements.

### 4.3. Shortcomings of This Study

The main shortcomings of this study were the following:

1. The OGTT was used as a reference diagnostic method for diabetes mellitus. The diagnostic threshold for 2-h PG was established in relation to the risk of diabetic retinopathy, a microvascular complication of diabetes mellitus [40]. However, glucose tolerance is influenced by complex interactions of factors, both physiological and environmental, which pose significant implications for clinical diagnosis and research. The considerations that could affect glucose tolerance and, therefore, the interpretation of the 2-h PG measurement, include the following:
  (a) Age and Gender: Age and gender are significant variables in glucose tolerance. Insulin sensitivity often decreases with age, resulting in higher 2-h PG levels [41]. Gender differences, particularly related to hormonal changes in females, could also affect glucose metabolism [42].
  (b) Diurnal Variability: Glucose tolerance is subject to diurnal variation, which could affect the 2-h PG test outcomes. Insulin sensitivity is generally higher in the morning than in the evening [43].
  (c) Physical Activity: Exercise improves insulin sensitivity and therefore could affect glucose tolerance tests. The timing and intensity of physical activity could have a direct influence on the 2-h PG results [44].
  (d) Dietary Patterns: Short-term and long-term dietary habits, including the macronutrient composition of the diet, may alter the body’s glucose and insulin response [45].
  (e) Stress and Emotional States: The acute stress response includes a transient rise in glucose levels as a result of catecholamine release, potentially affecting the 2-h PG test [46].
  (f) Medications: Certain medications like corticosteroids, antipsychotics, and diuretics affect glucose metabolism, thereby influencing 2-h PG test outcomes [47].
  (g) Genetic Factors: Genetic predispositions influence glucose tolerance, and not accounting for this introduce variability in the 2-h PG test [48].
2. The lognormal distributions and the KDE, as parameterized in Table 2, fitted only approximately to the NHANES datasets of FPG and HbA1c measurements. It is well known that biological measurands, such as FPG and HbA1c, may not follow textbook statistical distributions like normal or lognormal distributions. Numerous papers have noted the skewness or kurtosis in the distribution of metabolic variables, urging the use of flexible statistical models [49,50].

#### Related Statistical Software

All major general or Bayesian statistical software packages (OpenBUGS, Ver. 3.2.3, JASP^®^, Ver. 0.18.1, Matlab^®^, Ver. R2023b, NCSS^®^, Ver. 23.0.2, R, Ver. 4.3.1, SAS^®^, Ver.9.4M8, SPSS^®^, Ver. 29, Stan, Ver. 2.33.0, and Stata^®^ Ver. 18) include routines for Bayesian inference. The program presented in this work provides 29 different types of parametric and nonparametric plots. None of the above-mentioned programs provide this range of plots without advanced statistical programming.

## 5. Conclusions and Future Directions

The intricacies of the double-sigmoid curve and multimodal distributions introduce a new frontier in personalizing healthcare provision. While smoother relationships between measurements and Bayesian probability facilitate clinical interpretability, multimodal distributions might serve as sentinel indicators of underlying complexities or methodological shortcomings, thus providing a useful tool in the field of medical diagnosis.

As a pivotal next step, future research should aim to validate the utility and reliability of the Bayesian inference based method applied in this study through real-world clinical trials, in addition to extending its application to include more diagnostic modalities. The aim is to combine this approach with existing clinical protocols, thereby optimizing the diagnostic precision and consequently improving patient outcomes.

In addition to its potential for clinical applications, the computational tool developed for this study could hold considerable promise as an educational and research adjunct. By facilitating the analysis of Bayesian probabilities in disease diagnosis, it serves as an invaluable resource for both medical practitioners in training and experienced researchers in the field. Its modular design and user-friendly interface make it easily adaptable to various research settings and educational curricula, thereby accelerating the adoption and dissemination of Bayesian approaches in medical statistics and diagnostics.

## Figures and Tables

**Table 1:** The descriptive statistics of FPG and HbA1c datasets.

	Diabetic Patients		Nondiabetic Patients	
*n*	687		10,519	
Measurand (Units)	FPG (mg/dL)	HbA1c (%)	FPG (mg/dL)	HbA1c (%)
Mean	141.3	6.67	99.9	5.47
Median	124.0	6.30	99.0	5.50
Standard Deviation	54.0	1.57	10.1	0.38
Skewness	2.375	2.201	0.576	−0.058
Kurtosis	9.037	8.377	4.213	3.615
Correlation Coefficient	0.914		0.320	

**Table 2:** The settings of the program for Figure 3, Figure 4, Figure 5, Figure 6, Figure 7, Figure 8, Figure 9, Figure 10, Figure 11, Figure 12, Figure 13, Figure 14 and Figure 15.

	Diabetic Patients		Nondiabetic Patients	
Measurand (Units)	FPG (mg/dL)	HbA1c (%)	FPG (mg/dL)	HbA1c (%)
Parametric Distribution	Lognormal	Lognormal	Lognormal	Lognormal
Parametric Distribution Mean	141.3	6.67	99.9	5.47
Parametric Distribution SD	54.0	1.57	10.1	0.38
KDE Smoothing Bandwidth (SD units)	0.32	0.34	0.34	0.35
Correlation Coefficient	0.914		0.320	

## Data Availability

The data presented in this study is available at https://wwwn.cdc.gov/nchs/nhanes/default.aspx (accessed on 4 September 2023).

## Supplementary Materials

The following supporting information can be downloaded at: https://www.mdpi.com/article/10.3390/diagnostics13193135/s1, BayesianDiagnosis.nb: The program as a Wolfram Notebook. Available at https://www.hcsl.com/Tools/BayesianDiagnosis/BayesianDiagnosis.nb (accessed on 28 September 2023); Supplementary File: BayesianDiagnosis.nb can be found at https://zenodo.org/record/8414309 (accessed on 6 October 2023); Supplementary File S1 can be found at https://zenodo.org/record/8407804 (accessed on 6 October 2023); Supplementary File S2 can be found at https://zenodo.org/record/8414841 (accessed on 6 October 2023).

## Abbreviations

PDF	probability density function
CDF	cumulative distribution function
KDE	kernel density estimator
OGTT	oral glucose tolerance test
PG	plasma glucose
2-h PG	plasma glucose, measured two hours after oral administration of 75 g of glucose, during an OGTT
FPG	fasting plasma glucose
HbA1c	glycated hemoglobin A1c
NHANES	National Health and Nutrition Examination Survey

## Appendix A

### Appendix A.1. Formalisms and Notation

#### Appendix A.1.1. Tuples

**x**: an *n*-tuple ${(x}_{1},x_{2},\ldots ,x_{n})$

#### Appendix A.1.2. Parameters

*v*: prevalence of disease
*μ*, *m*: mean
*σ*, *s*: standard deviation
*ρ*, *r*: correlation coefficient
*k*: shape parameter
ϑ: scale parameter
*h*: nonparametric kernel density bandwidth

#### Appendix A.1.3. Functions

$f^{-1}$: the inverse of the function f
$\left|H\right|$: determinant of the matrix *H*
$P\left(A\right)$: probability of the event *A*
$P\left(A,B\right)$: conditional probability of the event *A* given the event *B*
$cov\left(X,Y\right)$: covariance of two jointly distributed random variables X and Y
$E\left[Z\right]$: expected value of a random variable Z
$ln\left(x\right)$: natural logarithm
$L\left(\theta |z\right)$: likelihood function of the parameter **θ** given the observed value *z* of the random variable *Z*
$L\left(\theta |z\right)$: likelihood function of the parameter **θ** given the observed values **z** of the random variable Z.
$l\left(\theta |z\right)$: loglikelihood function of the parameter **θ** given the observed value *z* of the random variable *Z*
$l\left(\theta |z\right)$: loglikelihood function of the parameter **θ** given the of observed values **z** of the random variable Z.
$p\left(x\right)$: probability mass function of a discrete variable *X*
$P_{Q}\left(k;q\right)$: the *k ^th^ q*-quantile of a random variable
$erf\left(z\right)$: error function
$erfc\left(z\right)$: complementary error function
$\Gamma \left(z\right)$: gamma function
$\gamma \left(z,x\right)$: incomplete gamma function
$Q\left(a,z\right)$: regularized incomplete gamma function
$\gamma \left(z,x_{0},x_{1}\right)$: generalized incomplete gamma function
$Q\left(z,x_{0},x_{1}\right)$: regularized generalized incomplete gamma function
$K\left(u\right)$: kernel function
$f\left(x\right)$: univariate PDF
$f\left(x;\theta \right),\;f\left(x|\theta \right)$: univariate PDF given the multivariate parameter **θ**
$f\left(x,y\right)$: bivariate PDF
$f\left(x,y;\theta \right),f\left(x,y|\theta \right)$: bivariate PDF given the multivariate parameter **θ**
$F\left(x\right)$: univariate CDF
$F\left(x;\theta \right),F\left(x|\theta \right)$: univariate CDF given the multivariate parameter **θ**
$F\left(x,y\right)$: bivariate CDF
$F\left(x,y;\theta \right),\;F\left(x,y|\theta \right)$: bivariate CDF given the multivariate parameter **θ**

Definitions of the aforementioned functions are presented in Supplementary File S2.

### Appendix A.2. Bayes Theorem

For the purposes of our study, Bayes’ theorem is formulated as follows:

$$P\left(D,T\right)=\frac{P\left(T|D\right)P\left(D\right)}{P\left(T\right)}=\frac{P\left(T,D\right)P\left(D\right)}{P\left(T,D\right)P\left(D\right)+P\left(T|\bar{D}\right)\left(1-P\left(D\right)\right)}$$

where:

$P\left(D,T\right)$ represents the posterior probability of having the disease given the test results z.
$P\left(T,D\right)$ denotes the likelihood of obtaining the test results z given the presence of the disease.
$P\left(T|\bar{D}\right)$ denotes the likelihood of obtaining the test results z given the absence of the disease.
$P\left(D\right)\;$ is the prior probability or prevalence v of the disease.
$P\left(T\right)$ signifies the overall probability of the test results z.

Therefore, for the possibly multivariate parameter **θ**:

$$P\left(D,T\right)=\frac{L_{D}\left(\theta |z\right)v}{L_{D}\left(z|\theta \right)v+L_{\bar{D}}\left(z|\theta \right)\left(1-v\right)}=\frac{f_{D}\left(z|\theta \right)v}{f_{D}\left(z|\theta \right)v+f_{\bar{D}}\left(z|\theta \right)\left(1-v\right)}$$

where $L_{D}\left(\theta |z\right)$ and $f_{D}\left(z|\theta \right)$ denote the likelihood function and the PDF in the presence of the disease, while $L_{\bar{D}}\left(z|\theta \right)$ and $f_{\bar{D}}\left(z|\theta \right)$ denote the respective functions in the absence of the disease.

### Appendix A.3. Parametric Distributions

#### Appendix A.3.1. Normal Distribution

(a) Univariate

The univariate normal distribution or Gaussian distribution is a continuous probability distribution of a random variable *X*. The general form of its PDF is:

$$f_{N}\left(x;\mu ,\sigma \right)=\frac{e^{-\frac{1}{2}{\left(\frac{x-\mu }{\sigma }\right)}^{2}}}{\sigma \sqrt{2\pi }}$$

where the parameter *μ* is the mean of *X*, while *σ* is its standard deviation [52].

(b) Bivariate

The bivariate normal distribution or Gaussian distribution is a continuous probability distribution of two normally distributed random variables *X* and *Y*. The general form of its PDF is:

$$f_{N}\left(x,y;\mu_{X},\sigma_{X},\mu_{Y},\sigma_{Y},\rho \right)=\frac{e^{-\frac{1}{2\left(1-\rho^{2}\right)}\left(\frac{{\left(x-\mu_{X}\right)}^{2}}{{\sigma_{X}}^{2}}-\frac{2\rho \left(x-\mu_{X}\right)\left(y-\mu_{Y}\right)}{\sigma_{X}\sigma_{Y}}+\frac{{\left(y-\mu_{Y}\right)}^{2}}{{\sigma_{Y}}^{2}}\right)}}{2\pi \sigma_{X}\sigma_{Y}\sqrt{1-\rho^{2}}}$$

where $\mu_{X}$ and $\mu_{Y}\;$ are the means of *X* and *Y*, $\sigma_{X}$ and $\sigma_{Y}\;$ are their standard deviations, and ρ their correlation coefficient [52].

#### Appendix A.3.2. Lognormal Distribution

(a) Univariate

The univariate lognormal distribution is a continuous probability distribution of a random variable *X* whose logarithm is normally distributed. The general form of its PDF is:

$$f_{L}\left(x;m,s\right)=\frac{e^{\left(-\frac{1}{2}{\left(\frac{ln\left(x\right)-m}{\sigma }\right)}^{2}\right)}}{xs\sqrt{2\pi }}$$

where *m* is the mean and *s* the standard deviation of ln(X) [52].

If *μ* and *σ* are the mean and the standard deviation of *X,* we have:

$$\mu =e^{m+\frac{1}{2}s^{2}}$$

$$\sigma =\sqrt{e^{2m+2s^{2}}}$$

Therefore,

$$m=ln\left(\frac{\mu^{2}}{\sqrt{\sigma^{2}+\mu^{2}}}\right)$$

$$s=ln\left(1+\frac{\sigma^{2}}{\mu^{2}}\right)$$

$$f_{L}\left(x;\mu ,\sigma \right)=\frac{e^{\left(-\frac{1}{2}{\left(\frac{ln\left(x\right)-ln\left(\frac{\mu^{2}}{\sqrt{\sigma^{2}+\mu^{2}}}\right)}{ln\left(1+\frac{\sigma^{2}}{\mu^{2}}\right)}\right)}^{2}\right)}}{\sqrt{2\pi }xln\left(1+\frac{\sigma^{2}}{\mu^{2}}\right)}=\frac{e^{\left(\frac{{\left(2ln\left(x\right)-2ln\left(\mu \right)+ln\left(1+\frac{\sigma^{2}}{\mu^{2}}\right)\right)}^{2}}{8ln\left(1+\frac{\sigma^{2}}{\mu^{2}}\right)}\right)}}{x\sqrt{2\pi ln\left(1+\frac{\sigma^{2}}{\mu^{2}}\right)}}$$

(b) Bivariate

The bivariate lognormal distribution is a continuous probability distribution of two lognormally distributed variables *X* and *Y*. If $m_{X}$ and $m_{Y}\;$ are the means of $ln\left(X\right)$ and $ln\left(Y\right)$, $s_{X}$ and $s_{Y}\;$ their standard deviations, and r their correlation coefficient, the general form of its PDF is [52]:

$$f_{L}\left(x,y;m_{X},s_{X},m_{Y},s_{Y},r\right)=\frac{1}{d}e^{a}$$

where

$$a=\frac{1}{2}\left(\frac{-\left(ln\left(y\right)-m_{Y}\right)b-\left(ln\left(x\right)-m_{X}\right)c}{{s_{X}}^{2}{\sigma_{Y}}^{2}-r^{2}{s_{X}}^{2}{s_{Y}}^{2}}\right)\;b=\left(ln\left(y\right)-m_{Y}\right){s_{X}}^{2}-r\left(ln\left(x\right)-m_{X}\right)s_{X}s_{Y}\;c=\left(ln\left(x\right)-m_{X}\right){s_{Y}}^{2}-r\left(ln\left(y\right)-m_{Y}\right)s_{X}s_{Y}\;d=2\pi xy\sqrt{{s_{X}}^{2}{s_{Y}}^{2}-r^{2}{s_{X}}^{2}{s_{Y}}^{2}}$$

We have

$$r=\frac{\mu_{X}\mu_{Y}}{\sigma_{X}\sigma_{Y}}\left(-1+e^{\rho \sqrt{ln\left(1+\frac{{\sigma_{X}}^{2}}{{\mu_{X}}^{2}}\right)ln\left(1+\frac{{\sigma_{Y}}^{2}}{{\mu_{Y}}^{2}}\right)}}\right)\sqrt{ln\left(1+\frac{{\sigma_{X}}^{2}}{{\mu_{X}}^{2}}\right)ln\left(1+\frac{{\sigma_{Y}}^{2}}{{\mu_{Y}}^{2}}\right)}$$

where $\mu_{X}$ and $\mu_{Y}\;$ are the means of *X* and *Y*, $\sigma_{X}$ and $\sigma_{Y}\;$ are their standard deviations and ρ their correlation coefficient.

Therefore,

$$f_{L}\left(x,y;\mu_{X},\mu_{Y},\sigma_{X},\sigma_{Y},\rho \right)=\frac{e^{\frac{ab+c}{d}}}{g}$$

where

$$a=-2\left(-1+e^{\rho \sqrt{ln\left(1+\frac{\sigma_{X}^{2}}{\mu_{X}^{2}}\right)ln\left(1+\frac{\sigma_{Y}^{2}}{\mu_{Y}^{2}}\right)}}\right)\left(ln\left(x\right)-ln\left(\frac{\mu_{X}^{2}}{\sqrt{\mu_{X}^{2}+\sigma_{X}^{2}}}\right)\right)$$

$$b=m_{X}m_{Y}\sqrt{ln\left(1+\frac{\sigma_{X}^{2}}{\mu_{X}^{2}}\right)ln\left(1+\frac{\sigma_{Y}^{2}}{\mu_{Y}^{2}}\right)}\left(ln\left(y\right)-ln\left(\frac{\mu_{Y}^{2}}{\sqrt{\mu_{Y}^{2}+\sigma_{Y}^{2}}}\right)\right)$$

$$c={\left(ln\left(y\right)-ln\left(\frac{\mu_{Y}^{2}}{\sqrt{\mu_{Y}^{2}+\sigma_{Y}^{2}}}\right)\right)}^{2}\sigma_{X}^{2}+{\left(ln\left(x\right)-ln\left(\frac{\mu_{X}^{2}}{\sqrt{\mu_{X}^{2}+\sigma_{X}^{2}}}\right)\right)}^{2}\sigma_{Y}^{2}$$

$$d=2\left({\left(-1+e^{\rho \sqrt{ln\left(1+\frac{\sigma_{X}^{2}}{\mu_{X}^{2}}\right)ln\left(1+\frac{\sigma_{Y}^{2}}{\mu_{Y}^{2}}\right)}}\right)}^{2}ln\left(1+\frac{\sigma_{X}^{2}}{\mu_{X}^{2}}\right)ln\left(1+\frac{\sigma_{Y}^{2}}{\mu_{Y}^{2}}\right)\mu_{X}^{2}\mu_{Y}^{2}-\sigma_{X}^{2}\sigma_{Y}^{2}\right)$$

$$g=2\pi xy\sqrt{-{\left(-1+e^{\rho \sqrt{ln\left(1+\frac{\sigma_{X}^{2}}{\mu_{X}^{2}}\right)ln\left(1+\frac{\sigma_{Y}^{2}}{\mu_{Y}^{2}}\right)}}\right)}^{2}ln\left(1+\frac{\sigma_{X}^{2}}{\mu_{X}^{2}}\right)ln\left(1+\frac{\sigma_{Y}^{2}}{\mu_{Y}^{2}}\right)\mu_{X}^{2}\mu_{Y}^{2}+\sigma_{X}^{2}\sigma_{Y}^{2}}$$

#### Appendix A.3.3. Gamma Distribution

(a) Univariate

The univariate Gamma distribution is a continuous probability distribution of a random variable *X*. The general form of its PDF is:

$$f_{G}\left(x;k,\vartheta \right)=\frac{1}{\Gamma \left(k\right)\vartheta^{k}}x^{k-1}e^{-\frac{x}{\vartheta }}$$

where *k* is a shape parameter, *θ* a scale parameter and $\Gamma \left(u\right)$ the gamma function [52].

The mean *μ* and the standard deviation *σ* of *X*, are calculated as following:

$$\mu =k\vartheta \;\sigma =k\vartheta^{2}$$

Therefore,

$$k=\frac{\mu^{2}}{\sigma^{2}}\;\vartheta =\frac{\sigma^{2}}{\mu }$$

and

$$f_{G}\left(x;\mu ,\sigma \right)=\frac{1}{\Gamma \left(\frac{\mu^{2}}{\sigma^{2}}\right){\left(\frac{\sigma^{2}}{\mu }\right)}^{\frac{\mu^{2}}{\sigma^{2}}}}x^{\left(\frac{\mu^{2}}{\sigma^{2}}-1\right)}e^{-\frac{x\;\mu }{\mu^{2}}}$$

(b) Bivariate

The bivariate Gamma distribution is a continuous probability distribution of two variables *X* and *Y*. The copula version of its PDF is:

$$f_{G}\left(x,y;k_{X},k_{Y},\vartheta_{X},\vartheta_{Y},\rho \right)=\frac{ab}{c}$$

where

$$a=e^{\left({erfc}^{-1}{\left(2Q\left(k_{\Upsilon },0,\frac{y}{\vartheta_{Y}}\right)\right)}^{2}+\frac{{\left(-\rho {erfc}^{-1}\left(2Q\left(k_{X},0,\frac{x}{\vartheta_{Χ}}\right)\right)+{erfc}^{-1}\left(2Q\left(k_{\Upsilon },0,\frac{y}{\vartheta_{\Upsilon }}\right)\right)\right)}^{2}}{-1+\rho^{2}}-\frac{y}{\vartheta_{\Upsilon }}-\frac{x}{\vartheta_{Χ}}\right)}\;b=x^{-1+k_{X}}y^{-1+k_{\Upsilon }}\vartheta_{\Upsilon }^{-k_{\Upsilon }}\vartheta_{Χ}^{-k_{X}}\;c=\sqrt{1-\rho^{2}}\Gamma (k_{X})\Gamma (k_{\Upsilon })$$

and $k_{X},k_{Y}$ are shape parameters, $\vartheta_{X},\vartheta_{Y}$ are scale parameters, and ρ the correlation coefficient of *X* and *Y.*

If $\mu_{X}$ and $\mu_{Y}\;$ are the means of *X* and *Y*, $\sigma_{X}$ and $\sigma_{Y}\;$ their standard deviations, and ρ their correlation coefficient, it can be shown that:

$$f_{G}\left(x,y;\mu_{X},\mu_{Y},\sigma_{X},\sigma_{Y},\rho \right)=\frac{ab}{c}$$

where

$$a=e^{\left({erfc}^{-1}{\left(2Q\left(\frac{\mu_{Y}^{2}}{\sigma_{Y}^{2}},0,\frac{y\mu_{Y}}{\sigma_{Y}^{2}}\right)\right)}^{2}+\frac{{\left(-\rho \;{erfc}^{-1}\left(2Q\left(\frac{\mu_{X}^{2}}{{\sigma X}^{2}},0,\frac{x\mu_{X}}{{\sigma X}^{2}}\right)\right)+{erfc}^{-1}\left(2Q\left(\frac{\mu_{Y}^{2}}{\sigma_{Y}^{2}},0,\frac{y\mu_{Y}}{\sigma_{Y}^{2}}\right)\right)\right)}^{2}}{-1+\rho^{2}}-\frac{x\mu_{X}}{{\sigma X}^{2}}-\frac{y\mu_{Y}}{\sigma_{Y}^{2}}\right)}\;b=x^{\left(-1+\frac{\mu_{X}^{2}}{{\sigma X}^{2}}\right)}y^{\left(-1+\frac{\mu_{Y}^{2}}{\sigma_{Y}^{2}}\right)}{\sigma X}^{-\frac{2\mu_{X}^{2}}{{\sigma X}^{2}}}\mu_{X}^{\frac{\mu_{X}^{2}}{{\sigma X}^{2}}}\mu_{Y}^{\frac{\mu_{Y}^{2}}{\sigma_{Y}^{2}}}\sigma_{Y}^{-\frac{2\mu_{Y}^{2}}{\sigma_{Y}^{2}}}\;c=\sqrt{1-\rho^{2}}\Gamma (\frac{\mu_{X}^{2}}{{\sigma X}^{2}})\Gamma (\frac{\mu_{Y}^{2}}{\sigma_{Y}^{2}})$$

### Appendix A.4. Copulas

If $\mu_{X}$ and $\mu_{Y}\;$ are the means of the variables *X* and *Y*, $\sigma_{X}$ and $\sigma_{Y}\;$ their standard deviations, and ρ their correlation coefficient, it can be shown that the bivariate PDFs of the other copulas of the program are defined as follows:

#### Appendix A.4.1. X: Normally Distributed—Y: Lognormally Distributed

$$f_{NL}\left(x,y;\mu_{X},\mu_{Y},\sigma_{X},\sigma_{Y},\rho \right)=\frac{e^{c}d}{g}$$

where

$$a={-\frac{2ln\left(y\right)-2ln\left(\mu_{Y}\right)+ln\left(1+\frac{\sigma_{Y}^{2}}{\mu_{Y}^{2}}\right)}{2\sqrt{2ln\left(1+\frac{\sigma_{Y}^{2}}{\mu_{Y}^{2}}\right)}}}^{2}-\frac{{\left(2ln\left(y\right)-2ln\left(\mu_{Y}\right)+ln\left(1+\frac{\sigma_{Y}^{2}}{\mu_{Y}^{2}}\right)\right)}^{2}}{8ln\left(1+\frac{\sigma_{Y}^{2}}{\mu_{Y}^{2}}\right)}$$

$$b=-\frac{{\left(\frac{2ln\left(y\right)-2ln\left(\mu_{Y}\right)+ln\left(1+\frac{\sigma_{Y}^{2}}{\mu_{Y}^{2}}\right)}{2\sqrt{2}\sqrt{ln\left(1+\frac{\sigma_{Y}^{2}}{\mu_{Y}^{2}}\right)}}\sqrt{ln\left(1+\frac{\sigma_{Y}^{2}}{\mu_{Y}^{2}}\right)}\mu_{Y}+\rho {\sigma_{Y}erfc}^{-1}\left(2Q\left(\frac{\mu_{X}^{2}}{\sigma_{X}^{2}},0,\frac{x\mu_{X}}{\sigma_{X}^{2}}\right)\right)\right)}^{2}}{ln\left(1+\frac{\sigma_{Y}^{2}}{\mu_{Y}^{2}}\right)\mu_{Y}^{2}-\rho^{2}{s\sigma }_{Y}^{2}}$$

$$c=a+b-\frac{x\mu_{X}}{\sigma_{X}^{2}}$$

$$d={\left(\frac{xm_{X}}{\sigma_{X}^{2}}\right)}^{\frac{\mu_{X}^{2}}{\sigma_{X}^{2}}}$$

$$g=xy\Gamma \left(\frac{\mu_{X}^{2}}{\sigma_{X}^{2}}\right)\sqrt{2\pi ln\left(1+\frac{\sigma_{Y}^{2}}{\mu_{Y}^{2}}\right)\left(1-\frac{\rho^{2}{s\sigma }_{Y}^{2}}{ln\left(1+\frac{\sigma_{Y}^{2}}{\mu_{Y}^{2}}\right)\mu_{Y}^{2}}\right)}$$

#### Appendix A.4.2. X: Lognormally Distributed—Y: Normally Distributed

$$f_{LN}\left(x,y;\mu_{X},\mu_{Y},\sigma_{X},\sigma_{Y},\rho \right)=\frac{e^{c}d}{g}$$

where

$$a={{erfc}^{-1}\left(2Q\left(\frac{\mu_{Y}^{2}}{\sigma_{Y}^{2}},0,\frac{y\mu_{Y}}{\sigma_{Y}^{2}}\right)\right)}^{2}-\frac{{\left(2ln\left(x\right)-2ln\left(\mu_{X}\right)+ln\left(1+\frac{\sigma_{X}^{2}}{\mu_{X}^{2}}\right)\right)}^{2}}{8ln\left(1+\frac{\sigma_{X}^{2}}{\mu_{X}^{2}}\right)}$$

$$b=-\frac{{\left({erfc}^{-1}\left(2Q\left(\frac{\mu_{Y}^{2}}{\sigma_{Y}^{2}},0,\frac{y\mu_{Y}}{\sigma_{Y}^{2}}\right)\right)\sqrt{ln\left(1+\frac{\sigma_{X}^{2}}{\mu_{X}^{2}}\right)}\mu_{X}+\rho \sigma_{X}\left(\frac{2ln\left(x\right)-2ln\left(\mu_{X}\right)+ln\left(1+\frac{\sigma_{X}^{2}}{\mu_{X}^{2}}\right)}{2\sqrt{2}\sqrt{ln\left(1+\frac{\sigma_{X}^{2}}{\mu_{X}^{2}}\right)}}\right)\right)}^{2}}{ln\left(1+\frac{\sigma_{X}^{2}}{\mu_{X}^{2}}\right)\mu_{X}^{2}-\rho^{2}\sigma_{X}^{2}}$$

$$c=a+b-\frac{y\mu_{Y}}{\sigma_{Y}^{2}}$$

$${d=\left(\frac{y\mu_{Y}}{\sigma_{Y}^{2}}\right)}^{\frac{m_{Y}^{2}}{s_{Y}^{2}}}$$

$$g=\left(\sqrt{2\pi }xy\Gamma \left(\frac{\mu_{Y}^{2}}{\sigma_{Y}^{2}}\right)\sqrt{ln\left(1+\frac{\sigma_{X}^{2}}{\mu_{X}^{2}}\right)}\sqrt{1-\frac{\rho^{2}\sigma_{X}^{2}}{ln\left(1+\frac{\sigma_{X}^{2}}{\mu_{X}^{2}}\right)\mu_{X}^{2}}}\right)$$

#### Appendix A.4.3. X: Normally Distributed—Y: Gamma Distributed

$$f_{NG}\left(x,y;\mu_{X},\mu_{Y},\sigma_{X},\sigma_{Y},\rho \right)=\frac{e^{\left({{erfc}^{-1}\left(2Q\left(\frac{\mu_{Y}^{2}}{\sigma_{Y}^{2}},0,\frac{y\mu_{Y}}{\sigma_{Y}^{2}}\right)\right)}^{2}-\frac{{\left(x-\mu_{X}\right)}^{2}}{2\sigma_{X}^{2}}+\frac{{\left(x\rho -\rho \mu_{X}+\sqrt{2}{\sigma_{X}erfc}^{-1}\left(2Q\left(\frac{\mu_{Y}^{2}}{\sigma_{Y}^{2}},0,\frac{y\mu_{Y}}{\sigma_{Y}^{2}}\right)\right)\right)}^{2}}{2\left(-1+\rho^{2}\right)\sigma_{X}^{2}}-\frac{y\mu_{Y}}{\sigma_{Y}^{2}}\right)}{\left(\frac{y\mu_{Y}}{\sigma_{Y}^{2}}\right)}^{\frac{\mu_{Y}^{2}}{\sigma_{Y}^{2}}}}{y\sigma_{X}\sqrt{2\pi \left(1-\rho^{2}\right)}\Gamma \left(\frac{y\mu_{Y}}{\sigma_{Y}^{2}}\right)}$$

#### Appendix A.4.4. X: Gamma Distributed– Y: Normally Distributed

$$f_{GN}\left(x,y;\mu_{X},\mu_{Y},\sigma_{X},\sigma_{Y},\rho \right)=\frac{e^{\left(-\frac{x\mu_{X}}{s_{X}^{2}}+\frac{{\left(y-m_{Y}+\sqrt{2}\rho {erfc}^{-1}\left(2Q\left(\frac{\mu_{X}^{2}}{\sigma_{X}^{2}},0,\frac{x\mu_{X}}{\sigma_{X}^{2}}\right)\right)\sigma_{Y}\right)}^{2}}{2\left(-1+\rho^{2}\right)\sigma_{Y}^{2}}\right)}{\left(\frac{x\mu_{X}}{\sigma_{X}^{2}}\right)}^{\frac{\mu_{X}^{2}}{\sigma_{X}^{2}}}}{x\sigma_{Y}\sqrt{2\pi \left(1-\rho^{2}\right)}\Gamma \left(\frac{\mu_{X}^{2}}{{s\sigma }_{X}^{2}}\right)}$$

#### Appendix A.4.5. X: Lognormally Distributed—Y: Gamma Distributed

$$f_{LG}\left(x,y;\mu_{X},\mu_{Y},\sigma_{X},\sigma_{Y},\rho \right)=\frac{e^{c}}{d}$$

where

$$a={\left(\frac{2ln\left(y\right)-2ln\left(\mu_{Y}\right)+ln\left(1+\frac{\sigma_{Y}^{2}}{\mu_{Y}^{2}}\right)}{2\sqrt{2ln\left(1+\frac{\sigma_{Y}^{2}}{\mu_{Y}^{2}}\right)}}\right)}^{2}-\frac{{\left(2ln\left(y\right)-2ln\left(\mu_{Y}\right)+ln\left(1+\frac{\sigma_{Y}^{2}}{\mu_{Y}^{2}}\right)\right)}^{2}}{8ln\left(1+\frac{\sigma_{Y}^{2}}{\mu_{Y}^{2}}\right)}\;b=-\frac{{\left(\left(-ln\left(y\right)+ln\left(\mu_{Y}\right)-\frac{ln\left(1+\frac{\sigma_{Y}^{2}}{\mu_{Y}^{2}}\right)}{2}\right)m_{Y}\sigma_{X}+\rho \left(x-\mu_{X}\right)\sigma_{Y}\right)}^{2}}{2\sigma_{X}^{2}\left(ln\left(1+\frac{\sigma_{Y}^{2}}{\mu_{Y}^{2}}\right)\mu_{Y}^{2}-\rho^{2}\sigma_{Y}^{2}\right)}\;c=a+b-\frac{{\left(x-\mu_{X}\right)}^{2}}{2\sigma_{X}^{2}}\;d=2\pi y\sigma_{X}\sqrt{ln\left(1+\frac{\sigma_{Y}^{2}}{\mu_{Y}^{2}}\right)\left(1-\frac{\rho^{2}\sigma_{Y}^{2}}{ln\left(1+\frac{s_{Y}^{2}}{\mu_{Y}^{2}}\right)\mu_{Y}^{2}}\right)}$$

#### Appendix A.4.6. X: Gamma Distributed—Y: Lognormally Distributed

$$f_{GL}\left(x,y;\mu_{X},\mu_{Y},\sigma_{X},\sigma_{Y},\rho \right)=\frac{e^{a+b}}{c}$$

where

$$a=-\frac{{\left(2ln\left(x\right)-2ln\left(\mu_{X}\right)+ln\left(1+\frac{\sigma_{X}^{2}}{\mu_{X}^{2}}\right)\right)}^{2}}{8ln\left(1+\frac{\sigma_{X}^{2}}{\mu_{X}^{2}}\right)}\;b=-\frac{{\left(\sqrt{ln\left(1+\frac{\sigma_{X}^{2}}{\mu_{X}^{2}}\right)}\mu_{X}\left(y-\mu_{Y}\right)-\rho \sigma_{X}\sigma_{Y}\left(\frac{2ln\left(x\right)-2ln\left(\mu_{X}\right)+ln\left(1+\frac{\sigma_{X}^{2}}{\mu_{X}^{2}}\right)}{2\sqrt{ln\left(1+\frac{\sigma_{X}^{2}}{\mu_{X}^{2}}\right)}}\right)\right)}^{2}}{2\sigma_{Y}^{2}\left(ln\left(1+\frac{\sigma_{X}^{2}}{\mu_{X}^{2}}\right)m_{X}^{2}-\rho^{2}\sigma_{X}^{2}\right)}\;c=2\pi x\sigma_{Y}\sqrt{ln\left(1+\frac{\sigma_{X}^{2}}{\mu_{X}^{2}}\right)\left(1-\frac{\rho^{2}\sigma_{X}^{2}}{ln\left(1+\frac{\sigma_{X}^{2}}{\mu_{X}^{2}}\right)m_{X}^{2}}\right)}$$

### Appendix A.5. Nonparametric Distributions

#### Appendix A.5.1. Histograms

A histogram is a graphical representation of the distribution of a tuple of observed values of a variable *X*. If *X* is a continuous random variable, the histogram is an estimate of the probability distribution of *X*.

#### Appendix A.5.2. KDEs

Given a tuple of independent and identically distributed observed values ${(x}_{1},x_{2},\ldots ,x_{n})$ of a random variable *X*, the univariate KDE ${\hat{f}}_{K}\left(x;n,h\right)$ is defined as [53]:

$${\hat{f}}_{K}\left(x;n,h\right)=\frac{1}{nh}\sum_{i=1}^{n}K\left(\frac{x-x_{i}}{h}\right)$$

where:

1. *n* is the number of the observed values of the variable.
2. *h* is the bandwidth, a positive scalar that determines the width and smoothness of the kernel.
3. $K\left(u\right)$ is the kernel function.

Given two tuples of independent and identically distributed observed values ${(x}_{1},x_{2},\ldots ,x_{n})$ and ${(y}_{1},y_{2},\ldots ,y_{n})$ of two random variables *X* and *Y*, the bivariate KDE $\hat{f}\left(x,y;n,h_{1},h_{2}\right)$ is defined as [53]:

$$\hat{f}\left(x,y;n,h_{1},h_{2}\right)=\frac{1}{n{\left|H\right|}^{\frac{1}{2}}}\sum_{i=1}^{n}K\left({\left(z-z_{i}\right)}^{T}H^{-1}\left(z-z_{i}\right)\right)$$

where

$$z=\left[\begin{array}{c} x\\ y \end{array}\right]\;z_{i}=\left[\begin{array}{c} x_{i}\\ y_{i} \end{array}\right]\;H=\left[\begin{array}{cc} h_{1}^{2} & \rho h_{1}h_{2}\\ \rho h_{1}h_{2} & h_{2}^{2} \end{array}\right]$$

and *ρ* is the correlation coefficient of the two tuples.

The program uses the Gaussian kernel function:

$$K\left(u\right)=\frac{1}{\sqrt{2\pi }}e^{-\frac{u^{2}}{2}}$$

(a) Univariate KDE

$$\hat{f}\left(x;n,h\right)=\frac{1}{nh}\sum_{i=1}^{n}\frac{1}{\sqrt{2\pi }}e^{-\frac{{\left(\frac{x-x_{i}}{h}\right)}^{2}}{2}}$$

(b) Bivariate KDE

$$\hat{f}\left(x,y;n,h_{1},h_{2}\right)=\frac{1}{2\pi {n\left|H\right|}^{\frac{1}{2}}}\sum_{i=1}^{n}e^{-\frac{1}{2}{\left(z-z_{i}\right)}^{T}H^{-1}\left(z-z_{i}\right)}$$

where

$$z=\left[\begin{array}{c} x\\ y \end{array}\right]\;z_{i}=\left[\begin{array}{c} x_{i}\\ y_{i} \end{array}\right]\;H=\left[\begin{array}{cc} h_{1}^{2} & \rho h_{1}h_{2}\\ \rho h_{1}h_{2} & h_{2}^{2} \end{array}\right]$$

Additional details concerning parametric and nonparametric distributions can be found in Supplementary File S2.

## Appendix B

### Appendix B.1. Software Availability and Requirements

*B.1.1.* Program name: Bayesian Diagnosis
*B.1.2.* Project home page: https://www.hcsl.com/Tools/BayesianDiagnosis/ (accessed on 28 September 2023)
*B.1.3.* Operating systems: Microsoft Windows, Linux, Apple iOS

Programming language: Wolfram Language

*B.1.4.* Other software requirements:
  1. For running the program: Wolfram Player^®^, freely available at: https://www.wolfram.com/player/ (accessed on 31 August 2023) or Wolfram Mathematica^®^.
  2. For editing the datasets: Wolfram Mathematica^®^.
*B.1.5.* System requirements: Intel^®^ i9™ or equivalent CPU and 32 GB of RAM
*B.1.6.6.* License: Attribution—Noncommercial—ShareAlike 4.0 International Creative Commons License

## Appendix C

### Appendix C.1. A Note about the Program

#### Appendix C.1.1. About the Program Controls

The program features a tabbed user interface, designed to streamline user interaction and facilitate navigation across its multiple modules and submodules.

The numerical settings are defined by the user with sliders. Sliders can be finely manipulated by holding down the alt key or opt key while dragging the mouse. They can be even more finely manipulated by also holding the shift and/or ctrl keys.

Dragging with the mouse rotates the three-dimensional plots, while dragging with the mouse while pressing the ctrl, alt, or opt keys zooms in or out.

#### Appendix C.1.2. Range of input parameters

*v*: 0.010–0.500
*μ*: 0.01–10,000.00
σ : 0.01–3000.00
*ρ*: −1.000–1.000
*h*: 0.01–2.00
*x*: 0.01–10,000.00
*y*: 0.01–100,00.00

#### Appendix C.1.3. Datasets

The software is preloaded with the following datasets:

*d1*: Quantitative measurements of the first measurand (FPG) from diseased individuals (diabetic patients), aged 40–60.

*d2*: Quantitative measurements of the second measurand (HbA1c) from diseased individuals (diabetic patients), aged 40–60.

*nd1*: Quantitative measurements of the first measurand (FPG) from nondiseased individuals (nondiabetic patients), aged 40–60.

*nd2*: Quantitative measurements of the second measurand (HbA1c) from nondiseased individuals (nondiabetic patients), aged 40–60.
