# Supplementary material for: A Bayesian Inference Based Computational Tool for Parametric and Nonparametric Medical Diagnosis
Source: Diagnostics (Basel). 2023 Oct 5;13(19):3135. doi: 10.3390/diagnostics13193135 (PMC10572594; doi:10.3390/diagnostics13193135)
Supplement: Supplementary file 1 [file diagnostics-13-03135-s001.zip › S1.pdf]

Diagnostics

A Bayesian Inference Based Computational Tool for Parametric and Nonparametric Medical Diagnosis

Supplement 1

Theodora Chatzimichail, MRCS [a], *Aristides T. Hatjimihail, MD, PhD* [b]

Hellenic Complex Systems Laboratory, Kostis Palamas 21, Drama 66131, Greece, [a]tc@hcsl.com, [b]ath@hcsl.com

Interface of the *Bayesian Diagnosis* program

Table of Contents

1. Introduction
2. System Requirements
3. Interface Overview
4. Input Parameters
5. Modules and Submodules
6. Output
7. Datasets
8. Notation and Abbreviations
9. Source Code
10. Conclusion
11. License

1. Introduction

The *Bayesian Diagnosis* program is a specialized computational tool developed to assist medical professionals and researchers in the field of diagnostics. Utilizing Bayesian inference, the program allows for the calculation, plotting, and comparison of Bayesian posterior probabilities of disease based on two distinct measurands of two diagnostic tests. The program is designed to be compatible with both parametric and nonparametric statistical methods, offering a versatile approach to medical diagnosis.

2. System Requirements

2.1. Processor

Intel Core i9® or equivalent CPU

2.2. System Memory (RAM)

32 GB+ recommended

2.3. Operating Systems

Microsoft Windows, Linux, Apple iOS

2.4. Software Requirements

Wolfram Player®, freely available at Wolfram Player or Wolfram Mathematica®.

3. Interface Overview

3.1. Tabbed Navigation

The program features an intuitive tabbed user interface, designed to streamline user interaction and facilitate effortless navigation across its multiple modules and sub-modules. Each tab is clearly labeled to correspond with its respective module, allowing for quick access to various functionalities.

3.2. Numerical Settings: Sliders

The program offers controls for numerical settings, which can be adjusted through sliders.

Fine Manipulation

For more precise control, hold down the '*alt*' or '*opt*' key while dragging the mouse. For even finer adjustments, also hold the '*shift*' and/or '*ctrl*' keys.

3.3. Non-Numerical Settings

These settings are controlled using buttons. Each button is labeled clearly to indicate its function.

3.4. 3D Plot Navigation

Rotation

Users can rotate the three-dimensional plots by dragging with the mouse.

Zoom

To zoom in or out, drag the mouse while pressing the '*ctrl*', '*alt*', or '*opt*' key

4. Input Parameters

The program allows users to input a variety of parameters, each with a specific range:

v: 0.010 – 0.500

μ: 0.01 – 10000.00

σ : 0.01 – 3000.00

ρ: –1.000 – 1.000

h: 0.01 – 2.00 (standard deviation units)

x: 0.01 – 10000.00

y: 0.01 – 10000.00

5. Modules and Submodules

The program is organized into three primary modules, each with multiple submodules:

5.1. Parametric Module:

Assumes two sets of parametric distributions.

5.2. KDE Module

Assumes two sets of Kernel Density Estimators (KDEs).

5.3. Parametric vs KDE Module

Assumes one set of parametric distributions and one set of KDEs.

Each module allows the user to define:

a. Prior probability of disease (prevalence)
b. Two sets of four univariate and two bivariate distributions
c. A pair of values (x, y) of the two measurands

6. Output

The program generates a comprehensive set of outputs to aid in the diagnostic process:

6.1. Plots

Posterior Probability of Disease Plots

These plots provide a visual representation of the posterior probabilities based on the selected distributions.

Probability Density Function (PDFs) Plots

These plots show the PDFs for each measurand and their combination. An option to overlay histograms on these plots is also provided.

Quantile-Quantile (Q-Q) Plots

Q-Q plots for each measurand in diseased and nondiseased populations show quantiles from the distribution of the measurand and the respective dataset against each other.

Probability-Probability (P-P) Plots

P-P plots for each measurand in diseased and nondiseased populations show the cumulative probabilities from the distribution of the measurand and the respective dataset against each other.

6.2.Tables

Statistics Tables:

These tables provide statistical measures like mean, median, standard deviation, skewness, kurtosis, log-likelihood, prior probability, and correlation coefficient for each set of distributions.

Probability of Disease Tables

These tables show the calculated probabilities of disease for each set of distributions and their differences.

7. Datasets

The software employs datasets sourced from the National Health and Nutrition Examination Survey (NHANES), an initiative by the Centers for Disease Control and Prevention, USA. These datasets are configurable and may be substituted by end users as necessary.

The software is preloaded with the following datasets:

d1: Quantitative measurements of the first measurand, specifically fasting plasma glucose, acquired from diseased individuals, specifically diabetics, and aged between 40 and 60 years.

d2: Quantitative measurements of the second measurand, specifically glycated hemoglobin, also acquired from diseased individuals, specifically diabetics, and aged between 40 and 60 years.

nd1: Quantitative measurements of the first measurand, fasting plasma glucose, acquired from nondiseased individuals, specifically nondiabetics, and aged between 40 and 60 years.

nd2: Quantitative measurements of the second measurand, specifically glycated hemoglobin, also acquired from nondiseased individuals, specifically nondiabetics, and aged between 40 and 60 years.

8. Source Code

8.1. Programming language

Wolfram Language

8.2. Software source code file format

Wolfram Notebook

8.3. Availability

The updated source code is available at:
https://www.hcsl.com/Tools/BayesianDiagnosis/BayesianDiagnosis.nb

8.4. License

The *Bayesian Diagnosis* program is licensed under the Creative Commons Attribution-NonCommercial-ShareAlike 4.0 International License.

9. Notation and Abbreviations

9.1. Parameters

ν : prevalence of disease

μ: mean

σ : standard deviation

ρ : correlation coefficient

h : nonparametric kernel density bandwidth

9.2. Functions

$P(disease)$: posterior probability of disease

9.3. Abbreviations

PDF: probability density function

KDE: kernel density estimator

OGTT: oral glucose tolerance test

FPG: fasting plasma glucose

HbA1c: glycated hemoglobin A1c

NHANES: National Health and Nutrition Examination Survey

10. Conclusion

The Bayesian Diagnosis program offers a robust and user-friendly interface for medical professionals and researchers to apply Bayesian inference in medical diagnosis. Its modular design and comprehensive output options make it a valuable tool in the field of medical statistics and diagnostics.

11. License

This document is licensed under the Creative Commons Attribution-NonCommercial-ShareAlike 4.0 International License.
