# Supplementary material for: A Bayesian Inference Based Computational Tool for Parametric and Nonparametric Medical Diagnosis
Source: Diagnostics (Basel). 2023 Oct 5;13(19):3135. doi: 10.3390/diagnostics13193135 (PMC10572594; doi:10.3390/diagnostics13193135)
Supplement: Supplementary file 1 [file diagnostics-13-03135-s001.zip › S2.pdf]

Diagnostics

A Bayesian Inference Based Computational Tool for Parametric and Nonparametric Medical Diagnosis

Supplement 2

Theodora Chatzimichail, MRCS [a], Aristides T. Hatjimihail, MD, PhD [b]

Hellenic Complex Systems Laboratory, Kostis Palamas 21, Drama 66131, Greece, [a]tc@hcsl.com, [b]ath@hcsl.com

Definitions and Formalities

Table of Contents

1. Functions
2. Nonparametric Distributions
3. Probability Plots
4. References
5. License

1. Functions

Inverse Function

The inverse function f^{-1} of a function f (also called the inverse of f) is a function that undoes the operation of f. Therefore:

$$f^{-1}\big(f(x)\big) = x$$

and

$$f\big(f^{-1}(y)\big) = y$$

Natural Logarithm

$$\ln(x) = \int_1^x \frac{1}{t} dt$$

Error Function

$$erf(x) = \frac{2}{\sqrt{\pi}} \int_0^x e^{-t^2} dt$$

Complementary Error Function

$$erfc(x) = 1 - erf(x)$$

Gamma Function

$$\Gamma(z) = \int_0^\infty t^{z-1} e^{-t} dt$$

for all complex numbers z, except the non-positive integers.

Incomplete Gamma Function

$$\gamma(z, x) = \int_x^\infty t^{z-1} e^{-t} dt$$

Regularized Incomplete Gamma Function

$$Q(z, x) = \frac{\gamma(z, x)}{\Gamma(z)}$$

Generalized Incomplete Gamma Function

$$\gamma(z, x_0, x_1) = \int_{x_0}^{x_1} t^{z-1} e^{-t} dt$$

Regularized Generalized Incomplete Gamma Function

$$Q(z, x_0, x_1) = \frac{\gamma(z, x_0, x_1)}{\Gamma(z)}$$

Probability Density Function

Univariate

The probability density function (PDF) is a statistical function that describes the likelihood of a continuous random variable taking on a particular value.

For a continuous random variable X, the PDF, denoted by $f(x)$, is defined as:

$$f(x) = \lim_{\Delta x \to 0} \frac{P(x \leq X < x + \Delta x)}{\Delta x}$$

where $P(x \leq X < x + \Delta x)$ is the probability that the random variable X falls within the interval $[x, x + \Delta x)$

Bivariate

The bivariate PDF is a statistical measure that describes the likelihood of two continuous random variables X and Y, taking on values x and y. It is denoted as $f_{X,Y}(x, y)$ and defined as:

$$f_{X,Y}(x, y) = \lim_{\Delta x, \Delta y \to 0} \frac{P(x \leq X < x + \Delta x, y \leq Y < y + \Delta y)}{\Delta x \Delta y}$$

where $P(x \leq X < x + \Delta x, y \leq Y < y + \Delta y)$ is the probability that the random variables X and Y fall within the intervals $[x, x + \Delta x)$ and $[y, y + \Delta y)$ respectively.

Cumulative Distribution Function

Univariate

The univariate cumulative distribution function (CDF) is closely related to the PDF and provides the cumulative probability for a random variable up to a specific value.

For a random variable X, the CDF, denoted by $F(x)$, is defined as:

$$F(x) = P(X \leq x) = \int_{-\infty}^{x} f(t)dt$$

where $f(x)$ is the PDF of the random variable.

The CDF is the integral of the PDF, and conversely, the PDF is the derivative of the CDF (when it exists):

$$f(x) = \frac{dF(x)}{dx}$$

Bivariate

The bivariate CDF is a function that describes the probability that the random variables X and Y simultaneously take on values less than or equal to x and y, respectively. It is denoted as $F_{X,Y}(x, y)$ and defined as:

$$F_{X,Y}(x, y) = \int_{-\infty}^{x} \int_{-\infty}^{y} f_{X,Y}(u, v)\, du\, dv$$

where $f_{X,Y}(x, y)$ is the PDF of the random variables.

Skewness

Skewness is a statistical measure that describes the asymmetry of a probability distribution about its mean. It quantifies the extent and direction of skew (departure from horizontal symmetry) in the data.

$$skewness(X) = \frac{\mathbb{E}[(X - \mu)^3]}{\sigma^3}$$

where X is a random variable and μ and σ are the mean and the standard deviation of X.

If $skewness(X) < 0$, the distribution is said to be left-skewed. If $skewness(X) > 0$, is said to be right-skewed. If $skewness(X) = 0$, the distribution is symmetric.

Kurtosis

Kurtosis is a statistical measure that quantifies how heavy the tails of a distribution are compared to a normal distribution.

$$kurtosis(X) = \frac{\mathbb{E}[(X - \mu)^4]}{\sigma^4}$$

where X is a random variable and μ and σ are the mean and the standard deviation of X.

If $kurtosis(X) = 3$, the distribution has the same kurtosis as the normal distribution (mesokurtic).

If $kurtosis(X) < 3$, the distribution is leptokurtic (light tails).

If $kurtosis(X) > 3$, the distribution is platykurtic (heavy tails).

Correlation Coefficient

The correlation coefficient $\rho_{X,Y}$ of two random variables $X$and Y, with means μ_X and μ_Y, is defined as:

$$\rho_{X,Y} = \frac{cov(X,Y)}{\sigma_X \sigma_Y}$$

where

$$cov(X,Y) = \mathbb{E}[(X - \mu_X)(Y - \mu_Y)]$$

Given two tuples $(x_1, x_2, \dots, x_n)$ and $(y_1, y_2, \dots, y_n)$, of independent and identically distributed observed values of two random variables $X$and $Y$with means μ_X and μ_Y, their correlation coefficient $\rho_{X,Y}$ is defined as:

$$\rho_{X,Y} = \frac{\sum_{i=1}^{n}(x_i - \mu_X)(y_i - \mu_Y)}{\sqrt{\sum_{i=1}^{n}(x_i - \mu_X)^2}\sqrt{\sum_{i=1}^{n}(y_i - \mu_Y)^2}}$$

The correlation coefficient quantifies the strength and direction of the linear relationship between $X$and Y. We have $-1 \le \rho_{X,Y} \le 1$. If $\rho_{X,Y} = 0$ it is implied that there is no linear dependency between the respective variables. If $\rho_{X,Y} = 1$ it signifies a perfect linear relationship between the variables. If $\rho_{X,Y} = -1$ it signifies a perfect negative linear relationship.

Likelihood and Loglikelihood Functions

The likelihood function of the possibly multivariate parameter $\boldsymbol{\theta}$ given the observed value x of the random variable $X$is defined as:

$$\mathcal{L}(\boldsymbol{\theta}|x) = f(x|\boldsymbol{\theta})$$

where $f(x|\boldsymbol{\theta})$ is the PDF of Xgiven $\boldsymbol{\theta}$.

The likelihood and loglikehood functions of $\boldsymbol{\theta}$, given the tuple $\mathbf{x} = (x_1, x_2, \dots, x_n)$ of independent and identically distributed observed values of a random variable X, are defined as:

$$\mathcal{L}(\boldsymbol{\theta}|\mathbf{x}) = \prod_{i=1}^{n} f(x_i|\boldsymbol{\theta})$$

$$l(\boldsymbol{\theta}|\mathbf{x}) = \sum_{i=1}^{n} ln\big(f(x_i|\boldsymbol{\theta})\big)$$

Quantiles

A quantile is a statistical term that refers to dividing a probability distribution into continuous intervals with equal probabilities or dividing a set of observed values of a random variable into subsets with the same probability mass $p_X(x)$.

$p_X(x)$ is a function that gives the probability that a discrete random variable is exactly equal to some value:

$$p_X(x) = P(X = x)$$

Specifically, the kth q-quantile of a probability distribution or a set of observed values is a numerical value that divides the data into q equal parts, such that exactly $\frac{k}{q}$ of the set of observed values or the underlying probability distribution is less than or equal to that value.

The kth q-quantile of a probability distribution with CDF $F(x)$ is given by [1]:

$$P_Q(k;q) = F^{-1}\left(\frac{k}{q}\right)$$

where $F^{-1}(x)$ is the inverse of $F(x)$.

2. Nonparametric Distributions

Histograms

A histogram is a graphical representation of the distribution of a set of observed values of a variable X. If X is a continuous random variable, the histogram is an estimate of the probability distribution X. To construct a histogram:

1. The range of the set of variable's observed values is divided into a set of bins.
2. The variable's observed values are sorted into each bin.
3. The number of variables observed values that fall into each bin are counted.

The height of each bar in the histogram corresponds to the count of variable's observed values in bin. The width of each bar corresponds to the width of the bin.

The Knuth method [2] is a Bayesian approach to determining the optimal number of bins for a histogram. It calculates the optimal bin width by maximizing a likelihood function, considering the variable's observed values as independently and identically distributed.

Given a tuple $(x_2, \ldots, x_n)$ of observed values of a variable X, we find the optimal bin edges $\mathbf{B} = (b_1, b_2, \ldots, b_k)$, by maximizing the following likelihood function:

$$\mathcal{L}(\mathbf{B}|X) = n!\left(\prod_{i=1}^{k}\frac{1}{n_i!}\right)\frac{1}{k^n}\frac{1}{(b_k - b_0)^n}$$

where n is the total number of observed values, k is the number of bins, n_i is the number of observed values in the ith bin, and b_0 and b_k are the minimum and maximum bin edges, respectively.

There are variations of histograms where the height of bars represents relative frequencies (proportions or probabilities) instead of raw counts. In such cases, the area under the histogram integrates to 1.

Kernel Density Estimators (KDEs)

Given a tuple of independent and identically distributed observed values $(x_1, x_2, \ldots, x_n)$ of a random variable X, the univariate KDE $\hat{f}_K(x;n,h)$ is defined as [3]:

$$\hat{f}_K(x;n,h) = \frac{1}{nh}\sum_{i=1}^{n} K\left(\frac{x - x_i}{h}\right)$$

where:

1. n is the number of the observed values of the variable,
2. h is the bandwidth, a positive scalar that determines the width and smoothness of the kernel. If h is too small, the estimate could be overly sensitive to noise in the data, leading to a "noisy" multimodal estimate. Conversely, if h is too large, the estimate could be overly smooth, potentially obscuring meaningful features in the data.
3. $K(u)$ is the kernel function, which satisfies the properties:
   3.1. $\int K(u)du = 1$
   3.2. $\int u^2 K(u)du < \infty$

Given two tuples of independent and identically distributed observed values $(x_1, x_2, \dots, x_n)$ and $(y_1, y_2, \dots, y_n)$ of two random variables X and Y, the bivariate KDE $\hat{f}(x, y; n, h_1, h_2)$ is defined as [3]:

$$\hat{f}(x, y; n, h_1, h_2) = \frac{1}{n|H|^{\frac{1}{2}}} \sum_{i=1}^{n} K\left((z - z_i)^T H^{-1} (z - z_i)\right)$$

where

$$z = \begin{bmatrix} x \\ y \end{bmatrix}$$

$$z_i = \begin{bmatrix} x_i \\ y_i \end{bmatrix}$$

$$H = \begin{bmatrix} h_1^2 & \rho h_1 h_2 \\ \rho h_1 h_2 & h_2^2 \end{bmatrix}$$

and ρ is the correlation coefficient of the two sets of datapoints.

A kernel function $K(u)$ could be conceptualized as a weighting mechanism in the context of kernel density estimation. For every observed value u_i the kernel function $K(u)$ superimposes a localized influence or "perturbation" centered at u_i. The magnitude and dispersion of this perturbation are governed by the properties of $K(u)$ and the bandwidth parameter h, respectively. Specifically, the amplitude of the perturbation at u_i is contingent upon the value of $K(u_i)$, while the scale or spread of this influence is modulated by h. This ensures that each observed value contributes to the overall density estimate in a manner that is both localized and smooth, with the degree of localization and smoothness being adjustable via the choice of $K(u)$ and h.

The program uses the Gaussian kernel function:

$$K(u) = \frac{1}{\sqrt{2\pi}} e^{-\frac{u^2}{2}}$$

3. Probability Plots

Quantile-Quantile (Q-Q) plot

A Q-Q plot is constructed by plotting the quantiles from a distribution and a tuple of observed values of a random variable, against each other. If the dataset comes from the theoretical distribution, the points in the Q-Q plot will approximately lie on the reference line $y = x$.

Probability-Probability (P-P) plot

A P-P plot is constructed by plotting the cumulative probabilities from a distribution and a tuple of observed values of a random variable, against each other. If the dataset comes from the theoretical distribution, the points in the P-P plot will approximately lie on the reference line $y = x$.

4. References

1. Hyndman, R.J.; Fan, Y. Sample Quantiles in Statistical Packages. *Am. Stat.* **1996**, *50*, 361–365, doi:10.1080/00031305.1996.10473566.
2. Knuth, K.H. Optimal Data-Based Binning for Histograms and Histogram-Based Probability Density Models. *Digit. Signal Process.* **2019**, *95*, 102581, doi:10.1016/j.dsp.2019.102581.
3. Gramacki, A. *Nonparametric Kernel Density Estimation and Its Computational Aspects*; Springer, 2017; ISBN 9783319716886.

5. License

This document is licensed under the Creative Commons Attribution-NonCommercial-ShareAlike 4.0 International License.
